# Radiation induced osteogenic sarcoma of the maxilla

**DOI:** 10.1186/1477-7819-3-49

**Published:** 2005-07-21

**Authors:** Om Prakash, Bipin T Varghese, Anita Mathews, Nileema Nayak, Krishnankutty Ramchandran, Manoj Pandey

**Affiliations:** 1 Department of Oral and Maxillofacial Surgery, Government Dental College, Thiruvananthapuram, 695011, Kerala, India; 2 Department of Surgical Oncology, Regional Cancer Centre, Thiruvananthapuram, 695011, Kerala, India; 3 Department of Pathology, Regional Cancer Centre, Thiruvananthapuram, 695011, Kerala, India; 4 Department of Imagiology, Regional Cancer Centre, Thiruvananthapuram, 695011, Kerala, India; 5 Department of Surgical Oncology, Jawaharlal Nehru Cancer Hospital and Research Centre, Idgah Hills, Bhopal, MP, India

## Abstract

**Background:**

Radiation induced sarcoma arise as a long term complication of radiation treatment for other benign or malignant conditions. They are of very rare occurrence in jaw bones and are even rarer in maxilla.

**Case presentation:**

Here we report a case of radiation induced sarcoma in a patient treated for squamous cell carcinoma of buccal mucosa with radiation who developed osteosarcoma of maxillary bone after six years. The patient was treated successfully with surgery.

**Conclusion:**

What should be the best treatment of radiation induced sarcoma is still debatable; however, surgery offers the best chance of cure. Role of reradiation and adjuvant chemotherapy needs to be further evaluated.

## Introduction

Osteosarcoma of the head and neck are rare tumours and constitute <10% of all osteosarcomas [1,2]. They involve mandible and maxilla with equal frequency [3]. Although the pathogenesis is unknown various predisposing factors have been proposed. These include preexisting bone lesions like bone cysts [4], osteogenesis imperfecta [5], osteochondroma [6], fibrous dysplasia [7], trauma, genetic factors, virus [8], and previous radiation [9]. Radiation induced sarcomas (RIS) are defined as tumours that develop after a latent period after radiation, with in the field of radiation, and have histological confirmation of a sarcoma [10]. Osteosarcoma is the commonest RIS in bone, however in head and neck area malignant fibrous histiocytoma is more common [11]. Post radiation osteogenic sarcoma of the facial bones have been reported in the bones with preexisting benign diseases such as fibrous dysplasia or Paget's disease. However only few cases have been reported in normal maxilla [3,12,13]. We report here a case of maxillary osteosarcoma occurring 6 years after treatment for squamous cell carcinoma of the buccal mucosa.

## Case Report

**Figure 1:**
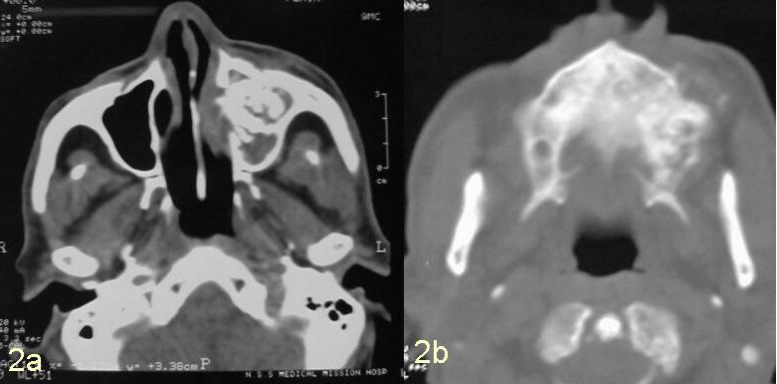
Computed tomographic scan showing lesion in maxillary antrum with destruction of maxilla (2a) and hard palate (2b).

A well built 76-year-old otherwise healthy male who was a known case of node negative squamous cell carcinoma of the left buccal mucosa, involving left retro molar trigone region, anterior tonsillar pillar and soft palate, and was treated with 60 Gy of external beam radiation using photons over 30 fractions delivered using cobalt 60 source in 1998, presented six years later in 2004 with the gradually progressive swelling and pain in left malar region for last 3 months. He developed numbness of left cheek and blurring of vision in left eye along with epiphora of left eye and nasal obstruction of left nare. There were episodes of epistaxis. He was a chronic chewer and smoker for more than 30 years but quit the habit after diagnosis of squamous cell carcinoma in 1998. Examination showed a bony swelling in left malar region measuring 2 × 3 cm, with diffused margins and blunting of infra orbital rim. Parasthesia of cheek left side, epiphora, blurring of vision in left eye, and obstruction of left nare were also present. Overlying skin appeared normal. Intra orally a total edentulous mouth and tender bony swelling in left maxillary alveolar region involving both buccal and palatal aspect was noted. Swelling was more pronounced over the buccal aspect and extended along whole of the anterior wall of maxilla. Computed tomographic scan demonstrated radiodense mass in left maxillary sinus with expansion of the anterolateral and posterolateral wall (figure 1). Tumor was seen to be infiltrating the orbital fat. At surgery the tumor was seen infiltrating ethmoid sinus, pterygoid muscles, lateral wall of nose and superomedial part of orbit. A local wide excision with left radical maxillectomy and orbital exenteration was carried out. The defect was grafted.

**Figure 2:**
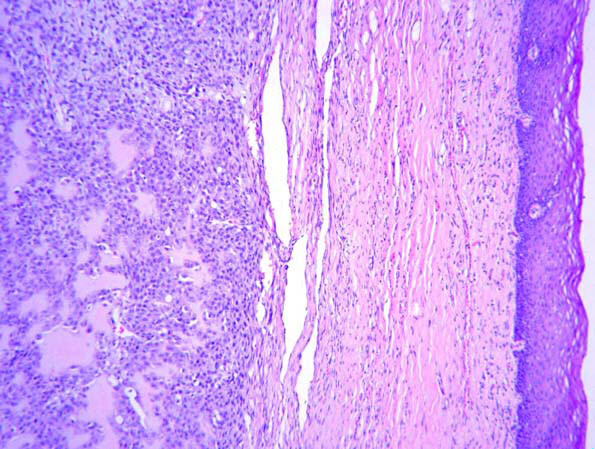
Photomicrograph showing a neoplasm composed of sheets of spindle cells having eosinophilic cytoplasm and hyper chromoatic nuclei (Hematoxylin & Eosin ×100).

Microscopic examination of the resected specimen demonstrated, a neoplasm composed of sheets of spindle cells having eosinophilic cytoplasm and hyper chromatic nuclei (figure 2). Laying of osteoid by the tumor cells was seen. Scattered mitotic figures were noted. The picture was consistent with osteogenic sarcoma of maxilla T3N0M0G1 AJCC stage III. The patient is disease free and on regular follow-up 6 months after the surgery.

## Discussion

Osteogenic sarcoma is the primary malignant tumour of the bone with predilection for long bones. Its occurrence in jaw bones is rare [1,2]. The average latent period between radiation treatment and development of sarcoma has been reported to be 4 to 30 years with average of 12.5 years [11]. The radiation dose varied from 25 to 110 Gy, with a median of 45 Gy [14]. Only few cases have been reported in a normal maxilla after radiation for benign or malignant disease of adjourning sites [3,11-13]. The exact mechanism of radiation induced sarcomas is not clear, these may occur after ortho (low energy) or mega voltage (high energy) radiation, however, with ortho voltage the dosage is lower and period is longer [15]. Development is also influenced by other known and yet unknown factors. It is suggested that the patients who harbor the mutation in tumor suppressor genes like p53 and ratinoblatoma gene (Rb) are more prone to develop these tumors. Furthermore children appear to be more susceptible than adults [16].

The treatment of osteogenic sarcoma of jaw irrespective of etiology includes radical surgery along with adjuvant chemotherapy and radiotherapy [3,17]. Survival after surgery alone is low however addition of adjuvant treatment show 8 year metastasis free survival rate of 60 to 70% [14,18] The factors associated with poorer prognosis include neural sensory alteration as a presenting symptom, increasing age of patients and surgical margins less than 5 mm [3,14].

What should be the ideal treatment for post radiation sarcoma is still debatable. Surgery appears to offer the best chance of cure. However as most of the osteosarcoma metastasize by hematogenous route, and hence there is a rational for addition of adjuvant chemotherapy [3,19]. Some authors recommend neoadjuvant chemotherapy before a definitive surgery is undertaken [20].

## Conclusion

The ideal treatment of radiation induced osteosarcoma still eludes surgeons. Surgery offers the best chances of cure provided a negative margin is achieved. Adjuvant chemotherapy should be offered to all cases as the hematogenous spread can occur. Some authors suggest use of neoadjuvant chemotherapy as this can help in achieving negative surgical margins.

## Competing interests

The author(s) declare that they have no competing interests.

## Authors' contributions

**OP: **Prepared the draft manuscript

**BTV**: was the main surgeon managing the case and helped in preparing the manuscript

**AM **and **NN**: did the histopathology and prepared the photomicrographs

**KR**: performed the imaging and provided the CT pictures

**MP**: helped in preparing the draft and edited the final manuscript, besides helping in patient management.
